# Medication by Proxy: The Devolution of Psychiatric Power and Shared Accountability to Psychopharmaceutical Use Among Soldiers in America’s Post-9/11 Wars

**DOI:** 10.1007/s11013-020-09673-7

**Published:** 2020-04-11

**Authors:** Jocelyn Lim Chua

**Affiliations:** University of North Carolina at Chapel Hill, Chapel Hill, USA

**Keywords:** Psychopharmaceuticals, Psychiatric medications, Sociality, Military personnel, United States Army

## Abstract

With the United States military stretched thin in the “global war on terror,” military officials have embraced psychopharmaceuticals in the effort to enable more troops to remain “mission-capable.” Within the intimate conditions in which deployed military personnel work and live, soldiers learn to read for signs of psychopharmaceutical use by others, and consequently, may become accountable to those on medication in new ways. On convoys and in the barracks, up in the observation post and out in the motor pool, the presence and perceived volatility of psychopharmaceuticals can enlist non-medical military personnel into the surveillance and monitoring of medicated peers, in sites far beyond the clinic. Drawing on fieldwork with Army personnel and veterans, this article explores collective and relational aspects of psychopharmaceutical use among soldiers deployed post-9/11 in Iraq and Afghanistan. I theorize this social landscape as a form of “medication by proxy,” both to play on the fluidity of the locus of medication administration and effects within the military corporate body, and to emphasize the material and spatial ways that proximity to psychopharmaceuticals pulls soldiers into relationships of care, concern and risk management. Cases presented here reveal a devolution and dispersal of biomedical psychiatric power that complicates mainstream narratives of mental health stigma in the US military.

## Introduction

As a light-wheel vehicle mechanic deployed to Iraq in 2006, Alex had a lot to keep track of during his 15- to 18-h workdays. He maintained and fixed vehicles, regularly went on convoys to Baghdad in search of repair parts, and pulled observation post-duty over a highway running through Fallujah. As his platoon’s Motor Sergeant, Alex was also responsible for the training, welfare and morale of his lower-ranking soldiers, duties that demanded a different kind of eye for detail. For instance, Alex told me he could infer a lot about the mental health of his soldiers by tracking their movements. Soldiers might need to arrange to get themselves on convoys in order to pick up medication or to meet the brigade psychiatrist at a larger base. “It would be, ‘Hey, someone needs to head to Baghdad,’ or ‘Somebody needs to head to Bagram,’ and we'd have to arrange a flight for them or get them on a truck. So you started picking up on what was what.” There were other ways of figuring out who needed looking out for. When Alex conducted “hooch checks”—inspections of sleep and work areas—opened drawers and dumped shave kits revealed which psychiatric medications were being taken and by whom. He made mental notes of it all. The signs were everywhere if you were properly attuned to them: “Let’s say one of your soldiers is changing into his ACUs (Army Combat Uniform) or into his coveralls, and *chichink,* a bottle would fall out on the floor. I’d just kind of pick it up, glance at it. ‘Did you drop this?’ Then you just look out for them after that.”

With the United States all-volunteer military stretched thin by prolonged and repeated deployments in the so-called “global war on terror,” military officials have embraced the easy dispensability of psychopharmaceuticals as an efficient means to enable more troops to remain “mission-capable” without dismissal from operational roles (Schneider, Bradley and Benedek 2007:685). The Army continues to lead all other military branches in the polypharmacy use of psychotropics, central nervous system depressant medications and opioids (Eide and Stahlman 2018:4). Beginning in the mid-1990s, the development of “cleaner” psychiatric medications, namely selective serotonin reuptake inhibitors (SSRIs), contributed to their incorporation into military garrison psychiatric practice at permanent military posts and bases, instep with their popular use in civilian practice (Pincus and Benedek^1998^; Schneider, Bradley and Benedek 2011). However, standard use of these medications in combat situations remained rare. The Army combat stress control field manual published in the late 1990s emphasized non-pharmacologic interventions, arguing that the effective soldier does not rely on pharmaceutical crutches, but rather is able to “master his own anxiety himself” (United States Department of the Army 1998:133).

This would shift with the post-9/11 wars in Iraq and Afghanistan in a manner that has arguably revolutionized the role of medications in the practice of US military operational psychiatry. In 2006, the US Department of Defense released for the first time official criteria guiding the authorized use of psychotropic medications for the treatment of ongoing disorders “in theater”—in the physical and tactical spaces of military operations including active combat (Assistant Secretary of the Defense 2006).^1^ As a hopeful panacea for exhaustion, strain and trauma, psychotropic medications have been critical to the post-9/11 Army’s efforts to address the embodied dilemmas of the chronic nature of America’s “forever war” (Filkins 2008). With the US’s longest global conflict running more than 18 years in Afghanistan, an unprecedented pattern of prolonged and repeated deployments has encouraged the use of psychiatric medications to stretch further an already taut military force.

Reliable data on psychopharmaceutical use among military service members in deployment is limited, since these medications are often prescribed outside of mental and behavioral health encounters, are rarely accessed in fixed locations and can circulate through informal practices that escape regulation (Chapelle and Lumley 2006:527; Assistant Secretary of Defense 2011). Yet, while the extent of the use of psychopharmaceuticals in deployment has been largely elusive to the surveillance and tracking mechanisms of military data systems, in interviews soldiers like Alex were quick to share their tacit, if not direct and intimate, knowledge of psychopharmaceutical use by others. Alex’s attunement to psychopharmaceutical use as sign of the mental health and overall state of his soldiers reveals the informal and subtle ways military personnel learn to read for the presence of psychotropic medications, and how they consequently become accountable to others in new ways through medication use.

In this article, I explore relational and collective aspects of psychopharmaceutical use among soldiers deployed in the post-9/11 wars in Iraq and Afghanistan. I theorize this social landscape as a form of “medication by proxy,” a term I use to play on the fluidity of the locus of medication administration and effects in contexts where medication use often has direct implications, not just for the individual on medication, but also for the military corporate body. This means that psychopharmaceutical use is often a site of collective negotiation and concern, in some cases to the point where the embodied and symbolic effects of medication are experienced across bodies. My use of the term “medication by proxy” also plays on the adjacent notion of proximity in order to emphasize the spatial, material and sensorial ways by which soldiers become attuned to the physical presence and use of psychotropic medications on deployment. Proximity to psychotropic medications within the intimate working and living conditions that characterize deployment for military personnel, and the perceived hazards this proximity often signifies for the collective, can consequently draw soldiers further into relationships of accountability and risk management in military institutions already governed by corporate logic and shared survival.

Scholars have recognized how the “pharmaceutical person” (Martin 2006) and the “pharmaceutical self” (Jenkins 2010b) are forged in relation to the proliferation of pharmaceutical use, in contexts where pharmaceutical markets now define a paradigm of “inherent illness” that enables the utilization of “drugs for life” (Dumit 2002, 2012), and where forms of governance shape the consumption of psychopharmaceuticals in defining possibilities for human life (Biehl 2004, 2013). In theorizing pharmaceutical subjectivity, scholars have also recognized the co-production of medication use and the intersubjective dynamics that shape the pharmaceutical self (Floersch 2004; Floersch et al. 2009; Jenkins and Carpenter-Song 2008; Kleinman 1988; Longhofer, Floersch and Jenkins 2003; Schlosser and Hoffer 2012). Medication by proxy extends these theorizations to consider how others around the pharmaceutical self are themselves oriented and attuned to the presence and use of medication, particularly in tightly integrated social and institutional contexts where medication effects may be borne by the collective. While the actual consumption of drugs matters centrally to theorizations of pharmaceutical subjectivity, medication by proxy also emphasizes how the *signifiers* of medication—the material and sensorial forms of medication’s presence and use—and the concreteness of medications in everyday life (Appadurai 1986; Ecks 2013; Lakoff 2005; van der Geest, Whyte and Hardon 1996) are as critical as the consumption of these medications to the formation of pharmaceutical subjectivity as a relational process.

On convoys and in the barracks, up in the observation post and out in the motor pool, proximity to psychopharmaceutical use can enlist non-medical military personnel across the ranks into the work of surveilling and managing medicated others. Soldiers like Alex become oriented and accountable to the use of medications by others in ways that offer productive insight into the devolution and dispersal of psychiatric power into sites, relations and practices that exceed the space of the clinic and the event of the provider encounter (Brodwin 2013; Estroff 1985; cf. Meyers 2013). Psychiatric power defines the “twin imperatives of care and constraint” (Brodwin and Velpry 2014) that shape diagnosis, treatment and subjectivities in psychiatry, power that is enabled by diverse institutions and assemblages in the post-asylum era (Foucault 1965, 2006). Military settings offer a relatively understudied and critical site for understanding how psychiatric power devolves from mental health professionals to soldiers, who now take on the work of monitoring their medicated peers, work which can be a constant and shifting task as operational environments and mission requirements—and thus potential threats and hazards—dynamically shift. This work may be enacted out of care for the soldier on medication, but also out of concern for the unit and for the collective success of “the mission.” As such, it may be taken up as felt duty, as it was for Alex, who naturally folded attunement to medication use into his paternal and disciplinary role as Motor Sergeant. But as we will see, in deployment this work is also shouldered as a form of risk management, where attunement to psychopharmaceutical use can evoke simultaneous and competing ideas of moral obligation, nuisance and threat.

The research that forms the basis of this article includes nineteen months of completed ethnographic fieldwork in North Carolina and Washington, DC, as well as six months of preliminary fieldwork I conducted in North Carolina and Virginia. Fieldwork, which is currently ongoing, has thus far included approximately 90 semi-structured interviews with active duty Army, Army National Guard and Army Reserve enlisted soldiers, officers and veterans who deployed post-9/11 to either Iraq or Afghanistan, as well as with military care providers. Interview participants have either themselves personally used or witnessed the use of psychopharmaceuticals by others while deployed. While I have interviewed Army personnel across the ranks, the majority of the interviews have focused on junior and senior enlisted soldiers of diverse occupations.^2^ Fieldwork has also included ethnographic observation at military health conferences, clinical trainings and veteran workshops, as well as focus groups with Army veterans.

## Pharmaceuticals, Sociality and the Military Corporate Body

Anthropologists and other scholars have recognized and explored the social relations through which pharmaceuticals are transacted, and the social dimensions of medication use (Ecks 2013; Floersch 2004; Floersch et al. 2009; Jenkins and Carpenter-Song 2008; Whyte, van der Geest and Hardon 2002). They have documented “both the somatizing and socializing potentials of pharmaceutical care,” demonstrating that pharmaceuticals involve social landscapes that exceed the individual consuming them (Schlosser and Ninnemann 2012:7). Pharmaceuticals can reconfigure relations between people, even as they are used to produce truth about those relations (Biehl 2004; Ma 2012; Pinto 2011). Interpretation of pharmacologic interventions and their effects also involve deeply social and cultural processes, shaping how they are received, understood and refused (Etkin 1992; Jenkins 2010a, 2010b; Lester 2014; Longhofer, Floersch and Jenkins 2003; Petryna and Kleinman 2006).

In military settings, the social dimensions of pharmaceuticals are further shaped by the collectivist nature of the military corporate body, as well as by the biopolitical and disciplinary regimes of power that characterize modern military institutions (Foucault 1979). While modest in their presentation when compared to high-technology weaponry or combat protective equipment, medications are nevertheless key to the protective and therapeutic technologies “that intervene at the level of biology to marshal soldier bodies as manipulable ‘resources’ that can be kept alive and allowed to die” (MacLeish 2012:55). Authorized at least in part to prop up forces strained by historically unprecedented repeat and long deployments in the post-9/11 wars, stimulants, sleep aids and antidepressants alike have been used to, in the words of the Army Medical Department, “conserve the fighting strength.”

But unlike standard issue vaccinations or medications administered to soldiers—the weekly dose of the malarial prophylaxis mefloquine taken on “Malaria Mondays,” or the Motrin so commonly doled out at sick call for aches and pains that soldiers jokingly refer to it as “Vitamin M”—psychopharmaceuticals are typically customized in type and dosage, and are accessed and taken more discretely. Psychopharmaceuticals are also distinguished by their associations with ideas of volatility. This perceived volatility rests in part with how psychopharmaceuticals may be taken to signify underlying mental health disorder, and thus unfitness for war. Given the potential for adverse behavioral, neurobiological and psychological effects noted of psychotropic medications more generally (Carpenter-Song 2009; Floersch 2004), these medications also raise concern for drug effects and compromised soldiering in ways that antibiotics or vaccines, for example, generally do not. Military criminal cases such as that of Robert Bales, the former staff sergeant who murdered 16 Afghans in 2012 while on a cocktail of Army-prescribed medications, have galvanized public attention around the potential for psychopharmaceuticals to undermine soldier agency, moral accountability and self-control. Concern for the volatility of psychopharmaceuticals is further complicated by the ethical and legal principles of counterinsurgency doctrine in Iraq and Afghanistan, which have defined good soldiering as the use of “courageous restraint” to protect civilians in ambiguous contexts where lines between combatant and noncombatant are unclear (McChrystal 2009).

Soldiers become attuned to medication use because psychopharmaceuticals are important to how they think about safety and danger in post-9/11 counterinsurgency. Most have direct knowledge of psychotropic medication use in deployment as a double-edged sword, learning of drug benefits and hazards through personal experience, observation and rumor. They theorize in their own terms the ancient Greek notion of *pharmakon,* which characterizes drugs as both remedy and poison, life and death (Martin 2006). Soldiers discuss and advise one another on the positives and negatives of particular medications in relation to their day-to-day duties, and through a risk calculus informed by occupational hazards: Does the emotional blunting associated with SSRIs pose an impairment for a parachute rigger? What about for a human resource specialist managing paperwork from the relative safety of the base? Might a sleep aid create danger for the team if a gunner is woken up at two in the morning for an unscheduled night patrol? Calculations of risk and benefit in relation to psychopharmaceuticals are thus revealing of the broader geopolitics of empire, refracting the tactical and strategic nature of counterinsurgency doctrine through micro-level biopolitical practices of medication use.

Given the corporate nature of the military body—arguably enhanced in theater where operational performance and risk have heightened consequence—the questions of safety and danger provoked by *pharmakon* are borne across differently scaled bodies of units, teams and the Army as a whole, not only by the individual on medication or their prescribing care provider. As Alex’s observations with which this article opened suggest, soldiers often become attuned, and in turn accountable, to the psychopharmaceutical use of others in ways that are also shaped by military hierarchy and by the everyday labor of counterinsurgency. Enlisted officers like Alex honed a perceptivity to psychopharmaceutical use, understanding it to be one among many responsibilities for maintaining his unit’s “mission readiness.” This responsibility was informed by the big-brother obligation he already felt to keep an eye out for the junior enlisted soldiers under him. Among peers, concern for medication use often evolves out of intimate experiences of fear for safety: of being on patrol or operating weapons alongside someone medicated, for instance. The type of psychopharmaceutical used also shapes the moral contours of accountability among military personnel. Different types of medications—antidepressants versus stimulants, for instance—can sit very differently under the sign of mental and behavioral health, and in relation to concerns for risk and safety. As one behavioral healthcare provider explained it to me, while a soldier might be inclined to keep his SSRI use under wraps, another diagnosed with ADHD might proudly “wave his Adderall prescription around” to the envy of others. Thus, while much of the literature about mental health and the post-9/11 US military has generally emphasized mental health stigma as a pervasive characteristic of the military’s masculinist institutional culture and a barrier to treatment, psychotropic medication use reveals complex orientations to mental health that are not captured by ideas of “weakness” and disorder alone. Social landscapes of psychotropic medication use among soldiers, in which military personnel are pulled to actively negotiate and manage everyday use of medication around them, indicate more heterogeneous relationships to mental and behavioral health than mainstream narratives of stigma in the US military would suggest.

## Tacit and Direct Knowledge of Psychopharmaceutical Use Among Soldiers

A commissioned officer in the Army National Guard, Bradley deployed to Afghanistan in 2013 as a civil affairs team leader primarily responsible for liaising between the Army and Afghan authorities. When I asked him during an interview if he knew of any psychotropic medication use while he was deployed, he told me it was everywhere. “How did you know?” I asked him. “You just knew,” he answered plainly. “The task force I was with, we were all housed together,” he told me. “We all went in on missions together, everybody knows everybody better than most everyone’s family members.” In those circumstances, “everybody knows everything about everyone.” It also meant that lay knowledge about experience with medications—and not uncommonly, medications themselves—were freely shared.

If somebody was struggling with something or they couldn't sleep, you would often have somebody come down the hall, knock on your door, and say, “Hey man, I can't sleep.” And they come to find out, well this helps me sleep. Or you hear, “Man, I took Ambien last night and I woke up in the bathroom.” You have so many people that are that close together. Kind of having the same coping mechanisms. “I'm super stressed out and I can't sleep and I need sleep. So, can somebody throw me an Ambien?”

While degrees of forced intimacy are typically a function of military rank and occupation, and their material conditions can vary widely, by and large military personnel recognize that entry into service means the forfeiture of many aspects of personal space and privacy. Enlisted soldiers spoke of this being all the more the case while deployed in Iraq and Afghanistan where, depending on one’s location and occupation, regular access to toileting facilities might be a luxury and one’s living quarters for months on end might be shared tents, a shipping container converted into a 2-person housing unit, or simply an outdoor cot. But intimacy was more than just a matter of eating, sleeping, working and toileting together in tight quarters; it was also a matter of the military’s prevailing corporate ethos of mutual reliance, dependability and accountability. Psychotropic medication use entered into this social landscape, becoming present through these intimacies and reconfiguring them.

While Bradley claimed that the use of psychotropic medications was information freely shared between officers on his team while he was deployed, knowledge of the psychopharmaceutical use of others, particularly psychiatric medications, was more commonly discerned or deduced through material signs of medication presence, or through lay readings of behavioral changes and symptoms. Jonathan, an intelligence analyst who deployed to Iraq in 2006, first suspected his sergeant’s medication use when she was caught sleepwalking one night on base. He told me that it was then when soldiers in his unit starting talking: “I’m sure the stuff she was on was the hardcore stuff….[Later] it comes out that she was on Ambien and antidepressants and doing this.”

But there were other signs that came to their attention once the sleepwalking episode got the squadron talking. Given the unpredictable nature of her day-today movements on and outside the base, Jonathan’s sergeant was ordered to keep her medications on her at all times. Because of the distinctive rattle that the bottle of pills made in her pocket as she walked—a sound Jonathan remembers would announce her approach—he and others in the battalion took to calling her “Rattlesnake.” The rattling sound of tablets illustrates the material and sensory ways medication use can become evident to others in deployment. Psychiatric medications in particular are highly ambivalent in their meaning (Jenkins and Carpenter-Song 2005, 2008; Schlosser and Hoffer 2012), here serving as a warning to others even as they suggest stability of the soldier and treatment under the care of professionals. Given that the sergeant’s medication use coincided with her return to duty after a suicide attempt that was widely known to the unit, Jonathan explained how the rattling sound of pills rattled the platoon, prompting unease over the sergeant’s continued presence and the risks of someone sleep walking on a heavily armed base. For Jonathan, it also inspired quiet outrage over why medications were being used to keep a suicidal soldier downrange. While borne in part out of concern for safety, Jonathan’s outrage was also deeply principled, directed at the Army’s push to keep bodies downrange “at all cost.” Rattlesnake’s case prompted difficult questions that would ultimately lead Jonathan down the path of splitting from the Army: Did he want to continue to be part of a institution that uses medication to return someone to the very same conditions that encouraged their suicide attempt in the first place?^3^

The aural presence of Rattlesnake’s pills, a function of her being instructed to carry them on her at all times, is a telling illustration of the relationship between the mobility of psychotropic medications and the operational and doctrinal context of counterinsurgency in Iraq. Because military operations in Iraq and Afghanistan have lacked “rear” areas to which soldiers can be safely evacuated, stabilized and treated, as had been the case in Cold War-style conflict, deployed psychiatrists found that earlier doctrine on intervention and restorative care for combat operational stress reactions were misaligned with post-9/11 counterinsurgency conditions: “The idea that soldiers could be removed from the front lines and treated in the rear echelons no longer worked, because no true rear echelons exist in Iraq. It was now important to treat mood and anxiety disorders as far forward as possible” (Schneider et al. 2011:154). Thus, military advocates have argued that the dispensability of psychopharmaceuticals, and just as importantly, their mobility across the shifting frontlines of counterinsurgency, have been critical to enabling more troops to remain “mission-capable” (Schneider, Bradley and Benedek 2007:681).

The fluidity of psychopharmaceuticals in counterinsurgency settings—their portability, as well as the protean benefits and dangers posed by these medications as they travel across operational contexts—is important then to understanding proximity to psychopharmaceuticals in deployment, and the tacit and direct knowledge of medication use that soldiers commonly shared in interviews. In the next section, I elaborate on how the presence and use of psychopharmaceuticals among soldiers is an emergent site for the devolution and dispersal of biomedical psychiatric power, as soldiers themselves take up the work of monitoring, regulating and surveilling their medicated peers.

## “It was in the ether”

Sitting behind the large mahogany desk in his empty elementary school classroom, Amir begins by telling me how much he derived from his military service. As I adjust myself in the stiff wooden chair on the other side of the desk, I feel a bit like I’ve been sent to the principal. For one thing, Amir tells me, his “third-grade level” Arabic improved over the course of his three tours to Iraq during which, by default, he became the impromptu translator for his unit, alongside doing his daily jobs as a motor transport operator. More seriously, though, he told me that if he hadn’t enlisted, he wouldn’t be where he is today, pursuing a higher education degree while working in elementary education: “I was at-risk youth, myself and I was blessed enough to be in the right situation where I was guided toward the military.”

Even then, the military he entered was not the one he left after 15 years of service. During his early deployments to Iraq, the first of which was during the initial 2003 invasion, Amir told me that, as a junior enlisted soldier at the time, psychotropic medication use was not explicitly talked about. “Some people might not even wanna let you know, so it was hard to gauge. And as a male, if guys I knew were on something, they probably wouldn't have told us.” He explained that this was the prevailing sentiment in the Army early on in the conflicts. Today, he told me, there is far more awareness and acceptance of mental health issues and medication use in the military. Even then, Amir has distinct memories of a handful of senior officers and a few peers whom he knew—or strongly suspected—to be on medication during those early deployments. He recounted in particular a fellow junior enlisted soldier, Kara, who was very forthcoming about her Xanax prescription within the unit. “She would take her little blue pills in front of everybody, do her business,” Amir told me. “And she didn’t care. She knew she needed it. It helped her cope and operate.”

“She was a very high-strung person,” Amir continued. “You could piss her off in a heartbeat by saying the wrong thing. Then you’d be sitting there all day trying to recoup the damage you made. It was like I had a second wife there, you know what I’m saying?” Laughing, then stopping himself to assure me he wasn’t “being chauvinist,” he backpedalled, offering a different etiology to Kara’s “emotionality”: “It was the type of personality she had. Brash, unabashed when it came to telling you what she thought. And if you offended her, she’d let you know in the most true way she could. You could tell she was very emotional. The Xanax took the edge off of that.”

As motor transport operators responsible for supervising and operating trucks to transport cargo and people, Amir’s 8-person team worked together all hours of the day and night. Their duties were routine, even if their schedules staggered greatly from one day to the next: one morning they might have to be at the motor pool at 2 AM in the morning, getting ready to be out on the road two hours later; the next day, they might leave the base at 10 PM. Sometimes the team would be out in trucks for 48 or 72 h before returning back to base, which made for close confines and tight living. In those circumstances, proximity to others meant knowledge of medication use within the team, and in Kara’s case, careful monitoring for compliance. Indeed, as Amir explained, “the guys” in their unit became so attuned to Kara’s mood from one day to the next that they could tell when she had taken her medication, and more importantly, when she had not. “Oh yeah, like you could tell, we could tell when she was on her medication or not. We knew. The command knew. Everybody knew.” Short of seeing her taking her medication everyday, how did they know? I pushed.

The days when she wouldn’t take it, she’d be bitching about the command. And people do it. Even guys do it, bitch about the command. But you could still tell. In the mornings, when we would go to motor pool or before mission, something like that, when she was on the Xanax, she was happy, chipper. She was also more in tune with doing her job and just…I think she was more on task. Not griping about it.

Amir’s comments are striking, not only for what they reveal about gendered interpretations of emotionality in the day-to-day sociality of soldiering (Ben-Ari 1998; DeGroot 2007; Sassen-Levy and Amran-Katz 2007). They also illustrate how Amir and “the guys” came to interpret modulations in Kara’s emotions and behavior in direct causal relation to her medication. In monitoring Kara’s medication use *through* their interpretations of her shifting emotions, specific signs emerged as indicators of whether she had taken her medication that day or not: “Her smile. If she wasn’t smiling, she probably wasn’t on her meds or it was wearing off.” On days when they suspected this was the case, someone might jokingly suggest that she double check if she’d taken her meds. “She openly talked about it with us,” Amir recalled. “It was in the ether. It was out in the open.”

In deployment, psychopharmaceutical use by the individual can mobilize the collective into banal acts of surveillance, monitoring and compliance, where displays of emotion produce truth about the absence or presence of drugs and their neurochemical effects. For Amir and the other guys in the unit, this was work done, less out of concern for Kara’s well being, than for the benefit of the team. Amir made this corporate logic and its gendered nature clear when he told me that smoothing out his colleague’s edge was not only a matter of smoothing out interpersonal relationships in a workplace environment, or even of enabling Kara to stay deployed. Even more than this, keeping a brash woman medicated was a matter of everyone’s safety in a war zone: “For my safety, for everybody else’s, I viewed it as better for her.”^4^ By Amir’s calculus, keeping their fellow truck driver balanced and predictable, both in the motor pool and out on the roads, was critical to the safety of the team and mission.

## Medicated by Proxy: The Devolution of Psychiatric Power in Deployment

Even as psychotropic medications may be used to smooth out soldiers and “keep them in the fight,” as they were for Rattlesnake and Kara, they can also raise questions about impacts on performance and safety once taken. Concern for psychotropic medications in soldiering therefore cuts both ways, at once raising suspicion about fitness for duty while generating new concern for medication effects once administered. In some instances, unit members can even play critical roles in getting soldiers on and off medication, suggesting the direct pathways by which psychiatric power can devolve to non-medical military personnel who have direct stakes in the performance of medicated peers.

Matt is a former Special Forces engineer with 13 years of service that includes five combat tours to Iraq and Afghanistan. During these deployments, Matt served as part of an Operational Detachment Alpha, a small, versatile Special Forces team that traveled and worked closely to execute, in his words, “sensitive tactical operations.” Matt described his team as “a 12-man, self-sustaining, holistic unit that can go anywhere in the world and conduct operations.” ODAs are designed to be “a highly functional, cross-trained team when they’re working correctly. We all do each other’s jobs.”

Over the course of multiple, back-to-back tours together, Matt and his teammates came to be concerned for two of their own who, in Matt’s words, had become “overheated.” Using an abundance of metaphors and euphemisms without providing specific detail, Matt spoke of a “line” separating authorized violence from unauthorized killing, soldiering from barbarism. These two teammates, he told me, starting crossing this “line” too many times. So the team intervened with drugs: they confronted the two teammates and convinced them to ask their military provider about medication. Matt understood that in the case of high-value team members who had seen multiple, back-to-back combat tours, antidepressants could keep needed skills and bodies downrange. They could keep their cross-trained team—so mutually reliant on each other as they were—together.

But there was a cost, Matt told me. Just days after the two teammates were started on their SSRIs, it became clear to the others that the medications posed a problem. Matt insisted that, even in this short period of time, the team began to notice the SSRIs blunting the “killer’s instinct.” Evoking once again the notion of a “line” dividing necessary aggression and unauthorized violence, Matt said:

The problem is that I need you right *towards* that line, right? That’s where we’re expected to be, is right here on the edge. We all know where this line is. We have to be close to that line. But now when I give him the drugs, we’re way far back. When you’re back here, that’s when you get killed. And now I have questions about whether I can rely on you. Because that’s when you start losing your situational awareness, when you start losing your edge. When you start losing that killer’s instinct that you have to have to survive. Do they (the SSRIs) get them to calm down? Yes, but now they aren’t as effective as they were before.

Matt’s attribution of his teammates’ impaired soldiering to their SSRI use recalls the *pharmakon.* Illustrating the notion of medicine as both remedy and poison, Matt underscored the ambivalent and contradictory nature of substances and their manifold and context-dependent effects (Biehl 2010; Derrida 1981; Martin 2006). As Matt explained it, while the blunting effects of SSRIs might be little more than a nuisance for a college student studying for exams, they are fatal in a war zone. In the team’s experience, the SSRIs dangerously muted the two soldiers’ situational awareness and the sensory attunements they had gradually cultivated over the course of several deployments: their ability to sense IEDs, to “feel the presence of the enemy.” This threw off the whole team, and so the use of the SSRIs was stopped. Just as it was with getting them on the SSRIs in the first place, the decision to stop the team members’ medication use was a shared one.

While Matt’s account resonates in some ways with the devolution of control over timing and dosage of psychiatric medication use from providers to consumers (McKinney and Greenfield, 2010), here medication use departs from a model of personal decision-making and self-directed compliance. The team’s role in the eventual SSRI use of their peers was a collective decision, suggesting the relational and shared ways psychiatric medication use can be negotiated within the military corporate body. Short of administering the medications themselves, the attempt to calibrate two team members’ distance from “the line” through antidepressant use also suggests the devolution of psychiatric power to military peers: in this case, into the material and institutional contexts and social landscape of elite soldiering. This is a type of medication by proxy, wherein team members call upon a combination of lay and expert knowledge in order to shape, and perhaps coerce, the behavioral potentials of peers. The use of medications to alter peer behavior is here predicated on a particular fantasy of the titration of violence through precision medication, wherein SSRIs “do not act upon the person as a whole, but are targeted precisely to correct a specific anomaly” (Rose 2003: 410–411). The anomaly to be “corrected”—in this case, the legal and ethical indiscretions of “crossing the line”—is conceived as a particular type of problem, one whose euphemistic phrasing alludes to the violation of Rules of Engagement directives and international law.

But Matt’s story also suggests that medication by proxy—collective negotiations around psychopharmaceutical use in a highly-integrated team—can lead to the transference of medication effects across the corporate body. Indeed, it led to Matt’s team feeling medic*ated* by proxy: of the team itself being slowed down and rendered vulnerable by the loss of their fellow soldiers’ edge. As the group interprets the behavioral effects of SSRIs in the context of the particular sensorium and imperative to situational awareness that characterize their everyday life-world of counterinsurgency operations, the loss of a teammate’s edge is experienced as a matter of collective—and indeed, mortal—concern. Not only can psychotropic medication diffuse across soldier relations; so, too, can its lived effects.

### Experiments in Action

For Matt and his teammates, accountability to the medication use of others followed a corporate logic of performance and survival. So, too, for Shawn, who explained to me how he and a friend, a fellow corporal during their deployment during the Iraq invasion in 2003, experimented with medication together in a manner that drew them into a silent pact of mutual accountability. As a field artillery firefinder radar operator, Shawn was responsible for detecting combatant forces and alerting Army units using a firefinder, a highly specialized, mobile radar system that scans for incoming rocket, artillery and mortar fire. His experience of the Iraq war, he told me, was likely far different from those of the other soldiers I’d been speaking with who deployed later in conditions of counterinsurgency: Shawn had been among the 130,000 US troops involved in the major combat operations in the US-led invasion of Iraq. “There was no FOB (forward operating base) or nothing. We were sleeping in foxholes. And it wasn’t like…a lot of people say, ‘Well, I was in Iraq at this base.’ I never seen that. It was just moving from Kuwait all the way to Tikrit, just straight going through, blowing up everything.” Shawn was leaving for Kuwait to return home as then-President George Bush was declaring major combat operations over in Iraq, the now eponymous “mission-accomplished” banner sailing overhead on the aircraft carrier USS Abraham Lincoln.

Unable to sleep “’cause of the anxiety,” Shawn told me that he and his friend saw few options for help. That early in the war, he told me, “there was no such thing as mental health.” The only medical provider he had regular access to during deployment was a medic, and “he wasn’t there for that kind of psychiatric part, ‘cause, you know, we were supposed to be okay psychiatrically at that point (that early in the conflict). He wasn’t prepared for that.” Having been diagnosed with ADHD as a child and taken Adderall and Xanax for years prior to his enlistment, and with a mother who is a nurse, Shawn told me he was reasonably well-versed in different types of psychotropic medications and their effects. Desperate for something to help them get some sleep, he and his friend decided to take it upon themselves to source their own medication. It was when their platoon was coming through an Iraqi air force base that had been taken over by coalition forces that Shawn and his friend saw an opportunity: they raided a pharmacy on the installation. But they weren’t able to find the Xanax they had been hoping for, likely due to the special mission unit they had followed in. “The labels were written in English and in Arabic on the shelves,” Shawn recalled. “And the whole Xanax line of jars of medications was missing. Someone had gotten there before we did.” What they did find were sample packs of the antipsychotic, Thorazine. “I didn’t know that it had antipsychotic properties but I had heard from people that they used it in hospitals for psych patients so I knew it calmed people down,” Shawn explained. “So that’s what we were carrying out with.”

Shawn and his buddy were careful to keep their medication use between them, out of fear of being labeled weak and suffering disciplinary action. But they also knew they had to be exceedingly careful with a substance chemically unknown to them, so they took the medication at small dosages at first, and alternated schedule so that they could look after one another while just one of them was asleep on the medication: “In case, you know, combat kicked off, we could at least protect the other guy or whatever. Make sure that they’re in the hole in the ground, their foxhole.” By monitoring the other on the medication, and staying awake and alert in the event that the other needed protection in an indisposed state, Shawn and his friend not only accumulated empirical knowledge of how the other reacted to the medication and at what dose; each was also intimately accountable to the welfare and wellbeing of his fellow corporal in ways reconfigured by psychopharmaceutical use. In such a context, medication use was not—and could not—be a matter of the individual alone.

Shawn made a point to tell me that his hands had been tied in that situation: “That was the way we needed to do it at the time. At that point, that was the way the military was. Everyone looked down on the idea of going to a psychiatric doctor or anything.” Yet, Shawn made clear its potential ramifications for the corporate whole. Indeed, our interview was laced with hints of the continuing guilt he still feels for taking the risk, albeit a calculated one, even as he sometimes thinks that the sleep the Thorazine afforded them was probably a net benefit. In the end, he tells me, “Nothing ever happened. So we were okay with what we did.”

## Beyond “Stigma”: Relations of Obligation and Accountability

Recent studies have documented the historical prevalence of mental health stigma in the military, with the aim of changing knowledge and attitudes around mental health in order to reduce stigma and improve access to quality care (Acosta et al. 2014; Hoge, Auchterlonie and Miliken 2006; Pietrzak et al. 2009). Yet, the accounts offered here suggest that the construct of stigma itself is limiting to our understanding of how mental health and the use of psychotropic medications become situationally meaningful in deployment through questions of risk, safety, vulnerability and threat that are themselves dynamic, shifting in relationship to group interactions and mission demands. While psychotropic medication use can set soldiers apart from others as classical definitions of stigma suggest, it also draws soldiers together through shared concern for performance and survival, and through a military ethos of collective accountability.

If the concept of stigma permits us to see repression and secrecy, psychotropic medication use in the accounts shared here span a wide spectrum from discretion, to open secret, to performative display: from Kara’s showcasing of her blue pills; to careful team consultations in Matt’s story; to the quiet, discretionary pact between friends in Shawn’s case. These forms are shaped by different socialities—and their variable moral contours, relations of power and governing ideologies—among military personnel in a system of hierarchical rank. These socialities range from the forms of fictive kinship evoked by Bradley in his description of the intimacy with which he lived and freely shared information of psychopharmaceutical use with his officer peers, to the machine-like integration of Matt’s Special Forces team, to the fear and concern tamped down by hierarchical subordination in Jonathan’s case.

While mainstream approaches in biomedical psychiatry often regard medications as static objects that carry and produce distinct pharmacologic effects (Kirmayer and Raikhel 2009), by contrast these cases illustrate context-specific understandings of adverse effects. In theater, “routine” side effects like drowsiness, perhaps tolerable to someone with the time and ability to sneak in an afternoon nap, can pose significant operational hazards to self and others in deployment. Concerns for adverse medication effects also hinge more specifically on different military duties, and the institutional and material circumstances of proximity and sociality these duties involve, all in a delicately balanced and ever-changing landscape of vulnerability and risk. In Amir’s case, the team’s concern for keeping a fellow truck driver “balanced” emerged from the close confines in which they worked and were out on the roads on a daily basis, as well as from the perceived capacities for stability and temperance required to do the work of transporting people and cargo “outside the wire.” By contrast, for elite military personnel who are directly tasked to engage in violence, balancing out the problem soldiers on Matt’s team introduced new kinds of risk for the group. Soldiers develop a perceptivity to and concern for the medication use of others through dynamic assessments of risk informed by changing material environments, group structure and operational duties. In these social, institutional and operational landscapes, soldiers may become proxies of psychiatric power, negotiating, surveilling, monitoring and even coercing the medication use of others.

The diverse ways psychopharmaceuticals mobilize social relations among military personnel thus complicate prevailing narratives of mental health stigma in the US military. Medication and the psychotropically medicated soldier are not always problems. Indeed, for some, medication can offer powerful workarounds to hold teams together, or provide short-term solutions for getting by for a time: of catching the few hours of recuperative sleep needed to stay alert and alive, or to finish out a deployment. The ethnographic observation that the meanings, value and effects attributed to psychotropic medication use emerge situationally—that is, in relationship to specific mission requirements and group dynamics—suggests that, at least in deployment settings, stigma is not inherent to medications themselves or to medication use. Rather, military concern for psychopharmaceuticals is highly context-dependent, shaped by broader shared landscapes and perceptions of risk, vulnerability and safety that exceed exclusive focus and blame on the individual alone. Soldiers thus understand and work with the medication use of others in ways that are arguably more flexible and more deeply social than the Army’s own medical institutions, which have historically perpetuated stark categorization of individual service members as either “ready” or “ill” and unfit for duty.

## Conclusion

In circumstances of persistent and enduring conflict, the disciplines and technologies of psychology and psychiatry have been increasingly mobilized by Western militaries to enable their personnel to withstand longer and multiple deployments (Gray 2015: 112). As Howell (2011: 4) has described it, “[t]he high tempo of deployments in the War on Terror have been made possible, in part, through the use of the psy disciplines.” At the same time, mental health has become the primary idiom through which we make sense of war: “psychological injuries have taken center stage in the ways we talk about, digest, and engage with war and its consequences” (Hautzinger and Scandlyn 2014: 6; see also Kieran 2019; MacLeish 2019). The normalization of the use of pscyhopharmaceuticals in and at war is in keeping with these broader trends, reshaping both capacities for enduring war and the moral economy of war suffering.

It therefore bears reminding that the microdynamics in the social landscape of military psychopharmaceutical use explored here occur within a broader context of the global expansion of US military power, and the increasing reliance on psychotropic medications to provide psychological buoyancy to an all-volunteer force stretched thin by chronic war. As such, the devolution and expansion of psychiatric power beyond the clinic in this case is much more than spatial: it is eminently geopolitical, offering an opportunity to extend medical anthropological studies of psychiatry and psychopharmaceutical use into the theater of war, and into the institutional contexts and embodied tensions of what MacLeish (2015, 2019) calls “military biopolitics”: the predicament of being protected, enhanced and kept alive while allowed to die.

At the same time, it might be tempting to interpret surveillance and self-monitoring among soldiers with respect to *pharmakon* as yet another example of the broader trend in the neoliberalization of mental health in the US military. Training soldiers to serve as gatekeepers of their peers’ mental health and their own, whether through suicide prevention or resilience training programs, has become a cost efficient strategy to enlist soldiers into the work of surveillance, monitoring and prevention in mental health (Gray 2015; Howell 2011, 2012). But folding the complex social landscape of psychopharmaceutical use into a broader neoliberalization of the military both minimizes the significance of the materiality of these medications to shaping sociality, and the accidental ways medications come to reconfigure relations between soldiers. Medication by proxy among deployed military personnel provokes us to take seriously the materiality of environments and technologies (Brodwin 2010; Hardon and Sanabria 2017; Latour 1992; Schüll 2014), just as the medications themselves invite and implicate witnesses through accidental and everyday disclosures: the rattling pills in the pocket of a pair of ACUs and the bottles that fall to the floor, waiting to be picked up.

## Footnotes

1 In military jargon, a “theater” of war refers to the entirety of the land, sea and airspace area that is or that may become involved in operations of war. For the purposes of the arguments in this article and to reflect the spatial imaginaries used by my interlocutors, I use “theater” interchangeably with “deployment” or “deployment setting” to refer to the area involved in operations of war for the U.S. Army, and which includes both combat and noncombat (also known as “support”) operations.

2 Military hierarchy and rank are strictly codified and determine differences including job function, level of responsibility, rules for interpersonal communication, dress and pay. The two different ways of joining the US military—by enlisting and commissioning—mark a major division among military personnel: commissioned officers are considered the managers and planners, responsible for planning missions, giving orders and assigning enlisted soldiers tasks. Among enlisted soldiers, there are 13 Army ranks, which can be broken down into three groups: junior enlisted, non-commissioned officer ranks and senior non-commissioned officer ranks. In general, climbing the rank structure carries increasing levels of responsibility and demands greater levels of respect and deference.

3 It was the treatment of his platoon sergeant that first started Jonathan down the path of questioning his military service, a path that ultimately led to his political radicalization as an anti-war activist. For more on Jonathan’s story, see Chua 2016.

4 Amir’s comments about the dangers of women soldiers’ uncontained emotionality bear striking resemblance to constructs of women’s emotionality that have been used to argue against gender integration in the military. Many monographs have been written aimed at convincing readers that women should not be allowed in combat, for reasons including women’s emotionality. See, for example, Stephanie Gutmann’s *The Kinder, Gentler Military: Can America’s Gender-Neutral Fighting Force Still Win Wars?*

## References

[CR1] Acosta Joie, Becker Amariah, Cerully Jennifer, Fisher Michael, Martin Laurie, Vardavas Raffaele, Slaughter Mary Ellen, Schell Terry L. (2014). Mental Health Stigma in the Military.

[CR2] Appadurai Arjun (1986). The Social Life of Things: Commodities in Cultural Perspective.

[CR3] Assistant Secretary of Defense (2006). Policy Guidance for Deployment Limiting Psychiatric Conditions and Medications.

[CR4] Assistant Secretary of Defense (2011). Defense Health Board Recommendation Memorandum Pertaining to Psychotropic Medication Prescription Practices and Use and Complementary and Alternative Medicine Use.

[CR6] Ben-Ari Eyal (1998). Mastering Soldiers: Conflict, Emotions and the Enemy in an Israeli Military Unit.

[CR7] Biehl João (2004). Life of the Mind: The Interface of Psychopharmaceuticals, Domestic Economies and Social Abandonment. American Ethnologist.

[CR8] Biehl, João 2010 Human Pharmakon: Symptoms, Technologies, Subjectivities. *In* A Reader in Medical Anthropology: Theoretical Trajectories, Emergent Realities. Byron Good, Michael Fischer, Sarah Willen, and Mary-Jo Delvecchio Good, eds., pp. 213–231. Malden, MA: Wiley-Blackwell.

[CR9] Biehl João (2013). Vita: Life in a Zone of Social Abandonment.

[CR10] Brodwin Paul (2010). The Assemblage of Compliance in Psychiatric Case Management. Anthropology & Medicine.

[CR11] Brodwin Paul (2013). Everyday Ethics: Voices from the Front Line of Community Psychiatry.

[CR12] Brodwin Paul, Velpry Livia (2014). The Practice of Constraint in Psychiatry: Emergent Forms of Care and Control. Culture, Medicine and Psychiatry.

[CR13] Carpenter-Song Elizabeth (2009). Children’s Sense of Self in Relation to Clinical Processes: Portraits of Pharmaceutical Transformation. Ethos.

[CR14] Chapelle Wayne, Lumley Vicki (2006). Outpatient Mental Health Care at a Remote US Air Base in Southern Iraq. Professional Psychology: Research and Practice.

[CR5] Chua Jocelyn (2016). Fog of War: Psychopharmaceutical “Side Effects” and the United States Military. Medical Anthropology.

[CR15] DeGroot Gerard (2007). A Few Good Women: Gender Stereotypes, the Military and Peacekeeping. International Peacekeeping.

[CR16] Derrida Jacques, Johnson B (1981). Plato’s Pharmacy. Dissemination.

[CR17] Dumit Joseph (2002). Drugs for Life. Molecular Interventions.

[CR18] Dumit Joseph (2012). Drugs for Life: How Pharmaceutical Companies Define Our Health.

[CR19] Ecks Stefan (2013). Eating Drugs: Psychopharmaceutical Pluralism in India.

[CR20] Eide Richard, Stahlman Shauna (2018). Polypharmacy Involving Opioid, Psychotropic, and Central Nervous System Depressant Medications, Period Prevalence and Association with Suicidal Ideation, Active Component, US Armed Forces, 2016. Medical Surveillance Monthly Report.

[CR21] Estroff Sue (1985). Making It Crazy: An Ethnography of Psychiatric Clients in an American Community.

[CR22] Etkin Nina (1992). “Side Effects”: Cultural Constructions and Reinterpretations of Western Pharmaceuticals. Medical Anthropology Quarterly.

[CR23] Filkins Dexter (2008). The Forever War.

[CR24] Floersch Jerry (2004). The Subjective Experience of Youth Psychotropic Treatment. Social Work in Mental Health.

[CR25] Floersch Jerry, Townsend Lisa, Longhofer Jeffery, Munson Michelle, Winbush Victoria, Kranke Derrick, Faber Rachel, Thomas Jeremy, Jenkins Janis, Findling Robert (2009). Adolescent Experience of Psychotropic Treatment. Transcultural Psychiatry.

[CR26] Foucault Michel (1965). Madness and Civilization: A History of Insanity in the Age of Reason.

[CR27] Foucault, Michel 1979 Discipline and Punish: The Birth of the Prison. Trans. Alan Sheridan. New York: Vintage Books.

[CR28] Foucault, Michel 2006 Psychiatric Power: Lectures at the Collège de France, 1973–74. Trans. Graham Burchell. New York: Palgrave Macmillan.

[CR29] Gray Harriet (2015). The Trauma Risk Management Approach to Post-Traumatic Stress Disorder in the British Military: Masculinity, Biopolitics and Depoliticisation. Feminist Review.

[CR30] Gutmann Stephanie (2000). The Kinder, Gentler Military: Can America’s Gender-Neutral Fighting Force Still Win Wars?.

[CR31] Hardon Anita, Sanabria Emilia (2017). Fluid Drugs: Revisiting the Anthropology of Pharmaceuticals. Annual Review of Anthropology.

[CR32] Hautzinger Sarah, Scandlyn Jean (2014). Beyond Post-Traumatic Stress: Homefront Struggles with the Wars on Terror.

[CR33] Hoge Charles, Auchterlonie Jennifer, Miliken Charles (2006). Mental Health Problems, Use of Mental Health Services, and Attrition From Military Service After Returning from Deployment to Iraq or Afghanistan. JAMA.

[CR34] Howell Alison (2011). Madness in International Relations: Psychology, Security, and the Global Governance of Mental Health.

[CR35] Howell Alison (2012). The Demise of PTSD: From Governing through Trauma to Governing Resilience. Alternatives.

[CR36] Jenkins, Janis 2010a Introduction. *In* Pharmaceutical Self: The Global Shaping of Experience in an Age of Psychopharmacology. Janis Jenkins, ed., pp. 6-16. Santa Fe, NM: School for Advanced Research Press.

[CR37] Jenkins, Janis 2010b Psychopharmaceutical Self and Imaginary in the Social Field of Psychiatric Treatment. *In* Pharmaceutical Self: The Global Shaping of Experience in an Age of Psychopharmacology. Janis Jenkins, ed., pp. 17-40. Santa Fe, NM: School for Advanced Research Press.

[CR38] Jenkins Janis, Carpenter-Song Elizabeth (2005). The New Paradigm of Recovery From Schizophrenia: Cultural Conundrums of Improvement without Cure. Culture, Medicine and Psychiatry.

[CR39] Jenkins Janis, Carpenter-Song Elizabeth (2008). Stigma Despite Recovery: Strategies for Living in the Aftermath of Psychosis. Medical Anthropology Quarterly.

[CR40] Kieran David (2019). Signature Wounds: The Untold Story of the Military’s Mental Health Crisis.

[CR41] Kirmayer Laurence, Raikhel Eugene (2009). From Amrita to Substance D: Psychopharmacology, Political Economy, and Technologies of the Self. Transcultural Psychiatry.

[CR42] Kleinman Arthur (1988). The Illness Narratives: Suffering, Healing, and the Human Condition.

[CR43] Lakoff Andrew (2005). Pharmaceutical Reason: Knowledge and Value in Global Psychiatry.

[CR44] Latour Bruno, Bijker WE, Law J (1992). Where are the Missing Masses? The Sociology of a Few Mundane Artifacts. Shaping Technology/Building Society: Studies in Sociotechnical Change.

[CR45] Lester Rebecca (2014). Health as Moral Failing: Medication Restriction among Women with Eating Disorders. Anthropology & Medicine.

[CR46] Longhofer Jeffrey, Floersch Jerry, Jenkins Janis (2003). Medication Effect Interpretation and the Social Grid of Management. Social Work in Mental Health.

[CR47] Ma Zhiying (2012). When Love Meets Drugs: Pharmaceueticalizing Ambivalence in Post-Socialist China. Culture, Medicine and Psychiatry.

[CR48] MacLeish Kenneth (2012). Armor and Anesthesia: Exposure, Feeling, and the Soldier’s Body. Medical Anthropology Quarterly.

[CR49] MacLeish Kenneth (2015). The Ethnography of Good Machines. Critical Military Studies.

[CR50] MacLeish Kenneth (2019). How to Feel about War: On Soldier Psyches, Military Biopolitics and American Empire. BioSocieties.

[CR51] Martin Emily (2006). The Pharmaceutical Person. BioSocieties.

[CR66] McChrystal, Stanley 2009 Tactical Directive. Kabul, Afghanistan: International Security Assistance Force. http://www.nato.int/isaf/docu/official_texts/Tactical_Directive_090706.pdf.

[CR52] McKinney Kelly, Greenfield Brian (2010). Self-Compliance at “Prozac Campus”. Anthropology & Medicine.

[CR53] Meyers Todd (2013). The Clinic and Elsewhere: Addiction, Adolescents, and the Afterlife of Therapy.

[CR54] Petryna Adriana, Kleinman Arthur, Petryna Adriana, Lakoff Andrew, Kleinman Arthur (2006). The Pharmaceutical Nexus. Global Pharmaceuticals: Ethics, Markets, Practices.

[CR101] Pietrzak Robert H., Johnson Douglas C., Goldstein Marc B., Malley James C., Southwick Steven M. (2009). Perceived Stigma and Barriers to Mental Health Care Utilization Among OEF/OIF Veterans. Psychiatric Services.

[CR55] Pincus Simon, Benedek David (1998). Operational Stress Control in the Former Yugoslavia: A Joint Endeavor. Military Medicine.

[CR56] Pinto Sarah (2011). Rational Love, Relational Medicine: Psychiatry and the Accumulation of Precarious Kinship. Culture, Medicine and Psychiatry.

[CR100] Rose Nikolas, Ericson Richard V., Doyle Aaron (2003). The Neurochemical Self and its Anomalies. Risk and Morality.

[CR57] Sassen-Levy Orna, Amram-Katz Sarit (2007). Gender Integration in Israeli Officer Training: Degendering and Regendering the Military. Signs.

[CR58] Schlosser Allison, Hoffer Lee D. (2012). The Psychotropic Self/Imaginary: Subjectivity and Psychopharmaceutical Use Among Heroin Users with Co-Occurring Mental Illness. Culture, Medicine and Psychiatry.

[CR59] Schlosser Allison, Ninnemann Kristi (2012). The Anthropology of Pharmaceuticals: Cultural and Pharmacological Efficacies in Context. Culture, Medicine and Psychiatry.

[CR60] Schneider Brett, Bradley John, Benedek David (2007). Psychiatric Medications for Deployment: An Update. Military Medicine.

[CR61] Schneider Brett, Bradley John, Warner Christopher, Benedek David, Lenhart MK (2011). Psychiatric Medications in Military Operations. Textbooks of Military Medicine.

[CR62] Schüll Natasha Dow (2014). Addiction by Design: Machine Gambling in Las Vegas.

[CR63] United States Department of the Army (1998). Combat Stress Control in a Theater of Operations: Field Manual 8–51.

[CR64] van der Geest Sjaak, Whyte Susan, Hardon Anita (1996). The Anthropology of Pharmaceuticals: A Biographical Approach. Annual Review of Anthropology.

[CR65] Whyte Susan, van der Geest Sjaak, Hardon Anita (2002). Social Lives of Medicines.

